# RA and ω-3 PUFA co-treatment activates autophagy in cancer cells

**DOI:** 10.18632/oncotarget.22629

**Published:** 2017-11-22

**Authors:** Shenglong Zhu, Guangxiao Lin, Ci Song, Yikuan Wu, Ninghan Feng, Wei Chen, Zhao He, Yong Q. Chen

**Affiliations:** ^1^ State Key Laboratory of Food Science and Technology, Jiangnan University, Wuxi, China; ^2^ School of Food Science and Technology, Jiangnan University, Wuxi, China; ^3^ Wuxi Medical School, Jiangnan University, Wuxi, China; ^4^ National Engineer Research Center for Functional Food, Jiangnan University, Wuxi, China; ^5^ Beijing Innovation Center of Food Nutrition and Human Health, Beijing Technology and Business University, Beijing, China; ^6^ School of Medicine, Wake Forest University, Winston-Salem, North Carolina, USA; ^7^ Wuxi No. 2 Hospital, Jiangsu, P. R. China

**Keywords:** retinoic acid, ω-3 PUFAs, autophagy, breast cancer

## Abstract

Retinoic acid (RA), is a promising therapeutic agent for the treatment of breast cancer. However, metabolic disorders and drug resistance reduce the efficacy of RA. In this study, we found that RA and ω-3 polyunsaturated fatty acids (ω-3 PUFAs) synergistically induced cell death *in vitro* and *in vivo* and autophagy activation. Moreover, RA-induced hypercholesterolemia was completely corrected by ω-3 PUFA supplementation. In addition, we demonstrated that the effects of this combination on the autophagic flux were independent of the two major canonic regulatory complexes controlling autophagic vesicle formation. The treatment activated Gαq-p38 MAPK signaling pathways, which resulted in autophagy of breast cancer cells. Knockdown of Gαq or P38 expression prevented RA and ω-3 PUFAs from inducing autophagy. Data indicated that Gαq-p38activation was mediated by the co-activation of GPR40 and RARα in lipid rafts, rather than by the activation of GPR120, RARβ, or RARγ. The results of this study suggest that hyperlipidemic drug side effects may be ameliorated by the administration of ω-3 PUFAs. Thus, the therapeutic indexes of the corresponding drugs may be increased.

## INTRODUCTION

Retinoic acid (RA), the major bioactive metabolite of vitamin A, plays an important role in cell growth and differentiation [1]. Preclinical studies have shown that RA and its derivatives have significant anti-proliferative and pro-apoptotic effects in breast cancer. However, clinical trials in breast cancer patients have been disappointing; because of low efficacy, metabolic disorders, and drug resistance, especially in ER-negative breast cancer [2]. Metabolic disorders are key risk factors for breast cancer, and several clinical investigations have shown a significant association between metabolic syndrome and breast cancer in women [3, 4]. Therefore, unsatisfactory clinical results may be related to RA-induced hypertriglyceridemia and hypercholesterolemia.

Recently, statins and fibrates, which are most commonly used to treat metabolic dysfunction, have been shown to potentiate the anti-tumor activity of RA [5, 6]. This suggests that administering RA in combination with lipid-or cholesterol-lowering drugs may be an effective anti-tumor strategy in breast cancer treatment.

Dietary ω-3 polyunsaturated fatty acids (ω-3 PUFAs) consist mainly of docosahexaenoic acid (DHA; 22:6n-3), eicosapentaenoic acid (EPA; 20:5n-3), and α-linolenic acid (ALA; 18:3n-3). Epidemiological, clinical, and experimental studies have demonstrated that ω-3 PUFAs reduce the incidence and mortality of breast cancer and improve metabolic disorders [7, 8].

The molecular mechanisms by which RA exerts its anti-proliferative effects are not fully elucidated. However, it has been determined that RA binds to nuclear retinoic acid receptors (RARs). Recently, it has been reported that RA treatment causes Gαq activation and the formation of Gαq-RAR complexes in lipid rafts. Similarly, ω-3 PUFAs stimulate Gαq, which then interacts with either GPR120 or GPR40 [9-11]. Moreover, reports indicate that RAR down-regulation leads to RA resistance in breast cancer therapy, and that ω-3 PUFAs increase the expression of RA receptors and activate RA receptor signal pathways [12, 13]. These results suggest that the administration of ω-3 PUFAs may enhance RA signaling pathways and reduce RA resistance.

The aim of this study was to determine whether supplementing RA with ω-3 PUFAs enhanced its anti-tumor activity, reduced drug resistance, and improved metabolic disorders during breast cancer treatment.

## RESULTS

### Effects of ω-3 PUFAs and RA treatments on cell growth

**Figure 1:**
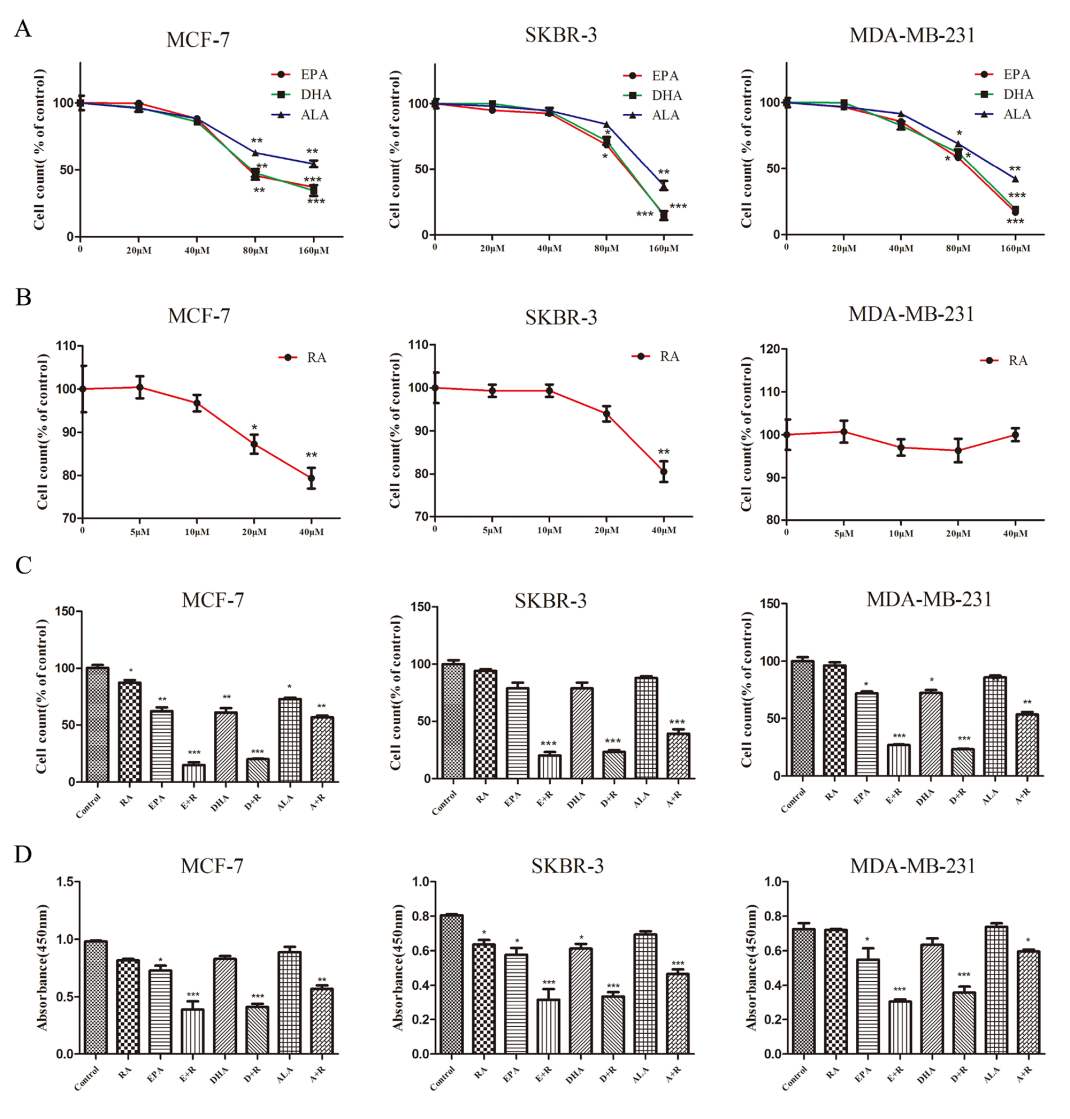
Growth inhibition in three breast cancer cell lines treated with RA and ω-3 PUFAs **(A-D)** cells treated with RA and/or ω-3 PUFAs for 72 h. (A): Cell counts after treatment with ω-3 PUFAs. (B): Cell counts after treatment with RA. (C): Cell counts after combination treatment. (D): Cell viability after combination treatment. Data are given as mean ± SEM. ^*^p < 0.05, ^**^p < 0.01, and ^***^p < 0.001.

Breast cancer is a heterogeneous disease which is classified into various subtypes based on ER, PR, and HER2 expression. Three human breast carcinoma cell lines (MCF-7, SKBR-3 and MDA-MB-231) were used in this study to determine the combined effects of RA and ω-3 PUFAs. Cells were treated with RA, ω-3 PUFAs or RA +ω-3 PUFAs for up to 3 days. As shown in Figure 1A and 1B and Supplementary Figure 1A and 1C, EPA, DHA, and ALA had no significant inhibitory effects at concentrations below 80μM. RA exhibited no significant cytotoxicity in MCF-7 and SKBR-3 cells at concentrations below 20μM. No inhibitory effects were detected in MDA-MB-231 cells, even at the highest RA concentration tested. This was attributed to these triple-negative breast cancer (TNBC) cells being more aggressive than the other cell lines and resistant to RA therapy [14]. Based on these results, 80μM ω-3 PUFAs and 20 μM RA were used in the combination treatments. Combination treatment significantly reduced cell counts and cell viability in all of the breast cancer cell lines. A maximal inhibition of 80%, compared to untreated cells, was achieved by day 3 in the combined RA and DHA or EPA treatment groups. Inhibition was significantly lower in the combined RA and ALA treatment group (Figure 1C and 1D and Supplementary Figure 1D). Overall, these results indicate that ω-3 PUFAs potentiated RA-induced cell death and increased RA sensitivity.

### RA and ω-3 PUFAs synergistically induce autophagy

Numerous reports been published concerning the relationship between autophagy and cancer progression and initiation. However, this relationship has not yet been completely clarified, and controversial results continue to be reported [15]. Previous studies have shown that both RA and ω-3 PUFAs induce autophagy in breast cancer cells [16, 17], although the phenomenon was not well demonstrated, nor the mechanism fully determined.

**Figure 2:**
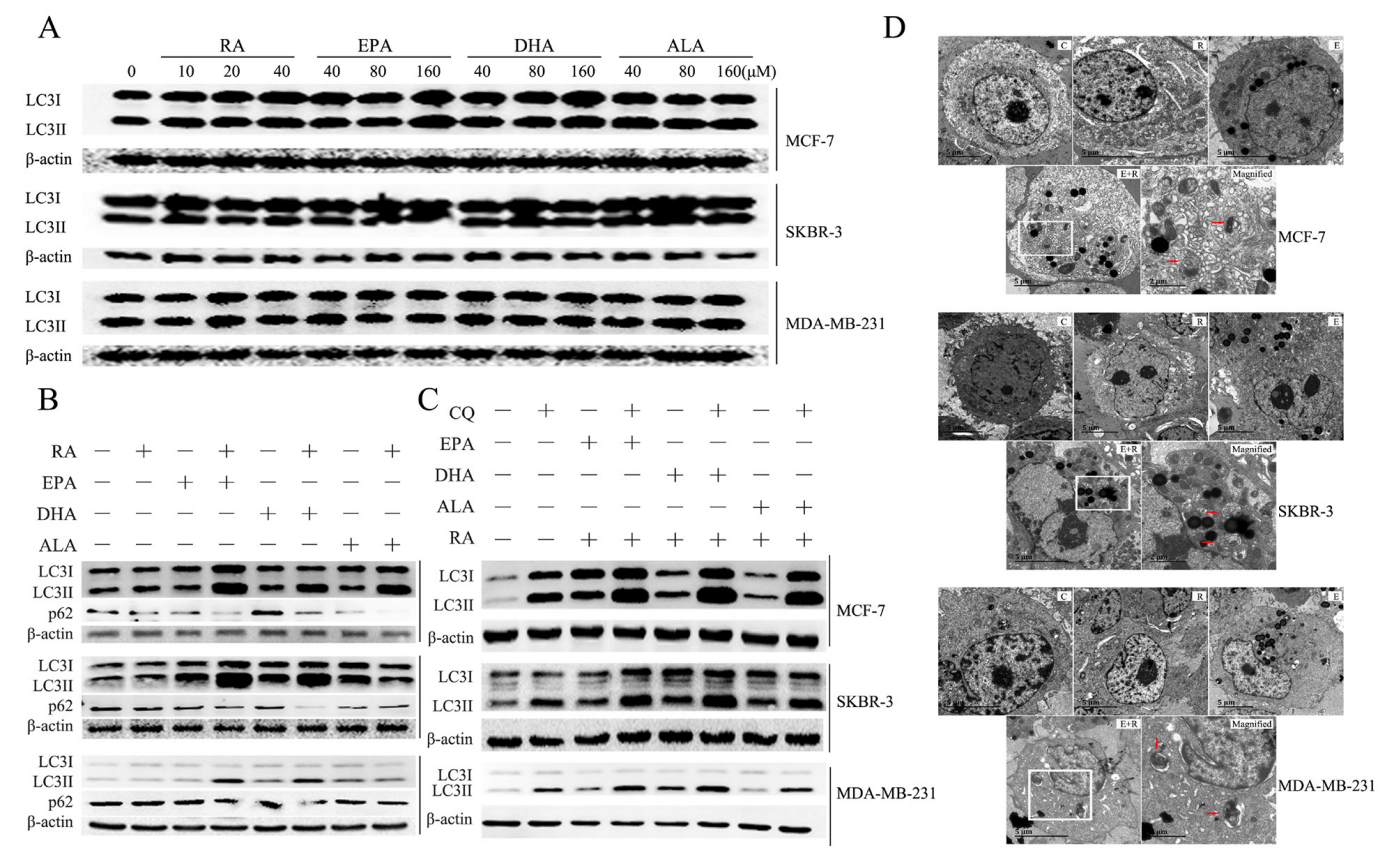
Autophagy induced by treatment with RA and ω-3PUFAs **(A)**: Cells were treated with RA or ω-3 PUFAs at indicated concentrations for 24h. Cell extracts were prepared and subjected to western blotting analysis. **(B)**: Cells were treated with RA(20μM) or ω-3 PUFAs(80μM) alone or in combination for 24h.Cell extracts were prepared and subjected to western blotting analysis. **(C)**: Cells were treated with RA(20μM) plus ω-3 PUFAs(80μM) with or without CQ(5μM) for 24h.Cell extracts were prepared and subjected to western blotting analysis. **(D)**: Cells were treated with RA(20μM) + EPA(80μM) for 24h prior to examination by TEM. Electron micrographs of MCF-7, SKBR-3, MDA-MB-231, control (C), RA-treated (R), EPA-treated (E), and RA-plus-EPA-treated (E+R) cells. Magnified images of the boxed regions of i showing autolysosomes orautophagosomes(magnified) (red arrows).

We speculated whether autophagy participated in the significant cell death and morphological change caused by RA and ω-3 PUFAS combination treatment. Unlike previous studies, we found that neither RA nor ω-3 PUFAs had a significant effect on LC3II expression (a biomarker of autophagosomes) in breast cancer cell lines (Figure 2A). However, the combination of RA and ω-3 PUFAs caused a significant increase in LC3II/β-actin in all of the breast cancer cell lines tested (Figure 2B)

The above results suggest that combination treatment upregulates the initiation of autophagy. However, the initiation and execution of autophagy is a highly dynamic process in mammalian cells. Therefore, monitoring autophagic flux is necessary for determining the rate of autophagy [18, 19]. Autophagic flux was measured by p62 degradation and by comparing LC3II/β-actin levels in combination treatment with or without CQ (by raising the lysosomal pH). These are two general methods for determining autophagic flux and are detailed elsewhere [20, 21]. Results showed that p62 were significantly decreased in combinational treatment in MCF-7, SKBR-3 and MDA-MB-231 and RA+ω-3 PUFAs +CQ treatment significantly increased cellular LC3II/β-actin levels, compared with cells treated with RA and ω-3 PUFAs (Figure 2B and 2C). The induction of autophagy was also determined by TEM, which is currently the gold standard for autophagy analysis. Autophagosome-like structures were readily detected in RA + EPA-treated cells (Figure 2D). In summary, combination treatment with RA and ω-3 PUFAs induced autophagy activation.

### Retinoic acid and ω-3 PUFAs modulate autophagic flux independent of mTOR and Beclin-1-complexes

Autophagy is tightly controlled by multiple signaling pathways, including the mTOR and Beclin-1 pathway [22, 23]. Because mTOR is a negative regulator of autophagy its activation leads to a suppression of autophagic vesicle formation. mTOR-phosphorylation at serine 2448 (S2448) and p70S6 kinase phosphorylation at threonine 389(T389), which is inhibited by rapamycin, are widely used as markers of mTOR activity [24].

**Figure 3:**
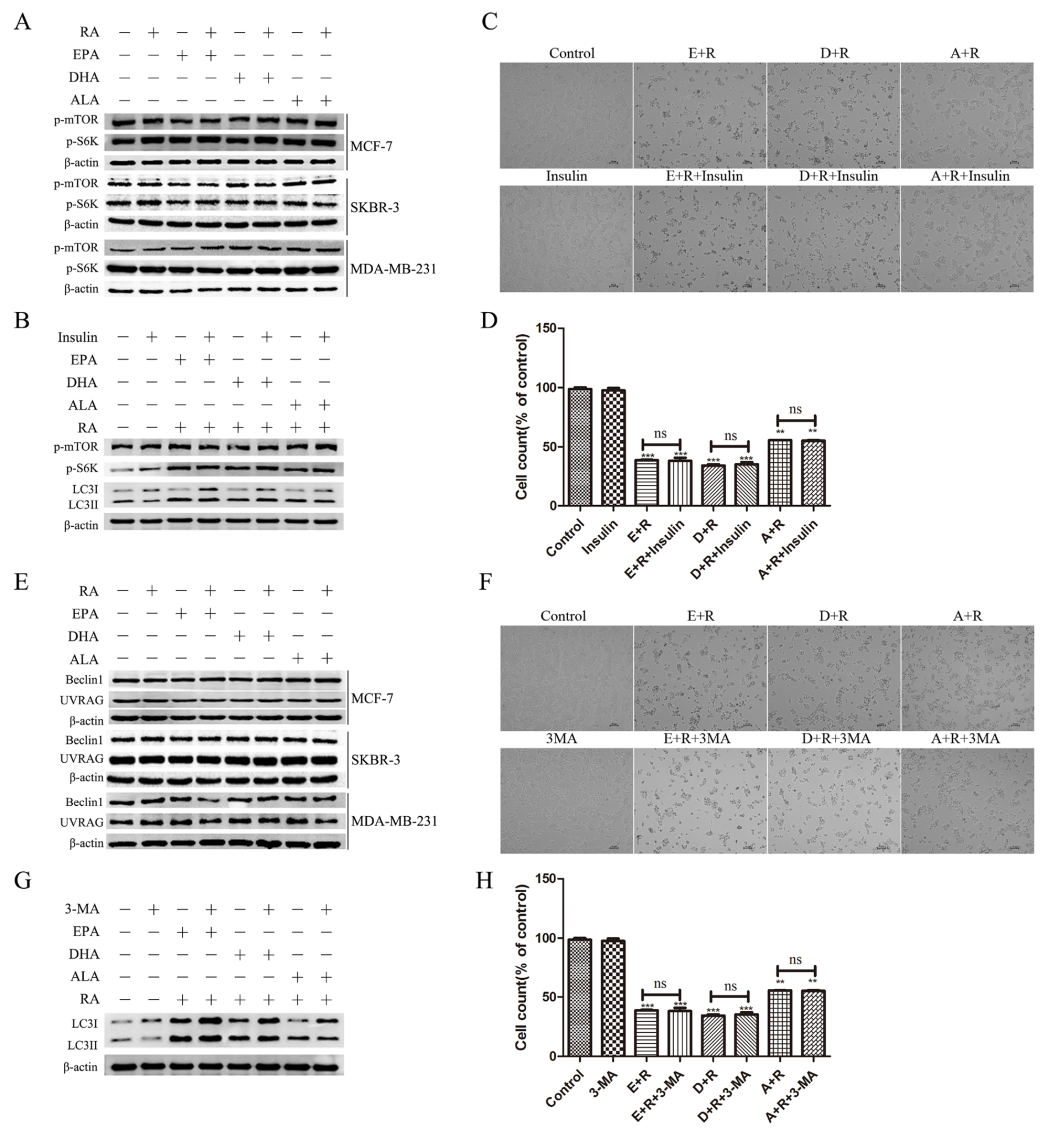
Autophagy induction by RA and ω-3 PUFA treatment is independent of two classical pathways **(A)**: Cells were treated with RA(20μM) or ω-3 PUFAs(80μM) alone or in combination for 24h. Cell extracts were prepared and subjected to western blotting analysis. **(B)**: Cells were treated with RA(20μM) +ω-3 PUFAs(80μM) with or without insulin (2μM) for 24h.Cell extracts were prepared and subjected to western blotting analysis. **(C)**: Cell morphology of MCF-7 cells treated with RA(20μM) + ω-3 PUFAs(80μM) with or without insulin (2μM) for 24h. **(D)**: Cell counts of MCF-7 cells treated with RA(20μM) plus ω-3 PUFAs(80μM) with or without insulin (2μM) for 24h. **(E)**: Cells were treated with RA(20μM) or ω-3 PUFAs(80μM) alone or in combination for 24h.Cell extracts were prepared and subjected to western blotting analysis. **(F)**: Cells were treated with RA(20μM) +ω-3 PUFAs (80μM) with or without 3-MA (5mM) for 24h.Cell extracts were prepared and subjected to western blotting analysis. **(G)**: Cell morphology of MCF-7 treated with RA(20μM) +ω-3 PUFAs(80μM) with or without 3-MA(5mM) for 24h. **(H)**: Cell counts of MCF-7 treated with RA(20μM) plus ω-3 PUFAs(80μM) with or without 3-MA(5mM) for 24h. Data are given as mean ± SEM. ^*^p < 0.05, ^**^p < 0.01, and ^***^p < 0.001.

Western blot analyses of the mTOR kinase substrate, the p70S6 kinase phosphorylation level (p-S6K), and phosphorylated mTOR (p-mTOR) were used to determine whether RA + ω-3 PUFAs induced autophagic flux by inhibiting mTOR activity. No differences were found between the amounts of p-mTOR and p-S6K present in any of the groups of the three cell lines tested (Figure 3A). Recent studies have established that insulin induces p70S6 kinase phosphorylation by mTOR [25, 26]. Therefore, we used insulin as an mTOR activator to determine whether mTOR activation can prevent the induction of autophagy by RA +ω-3 PUFAs. The results demonstrated that the addition of insulin had no significant effect on signal transduction or cell death (Figure 3B and 3D).

The second major protein complex that controls autophagic activity is that of Beclin-1. No significant differences in the concentrations of Beclin-1and UVRAG were determined between the cancer cell groups (Figure 3E). Furthermore, we determined whether 3-methyladenine (3-MA), which was widely used as a class III phosphatidylinositol 3-kinase (PtdIns3K) inhibitor, inhibited the induction of autophagy by RA +ω-3 PUFAs. Consistent with the results shown in Figure 3E, there were no significant differences between the samples treated with and without 3-MA (Figure 3F and 3H).

Overall, these results demonstrated that RA + ω-3 PUFAs induced autophagic flux were independent of mTOR and Beclin-1 complexes.

### Gαq-P38 pathways are required for the induction of autophagy by RA and ω-3 PUFAs

Mitogen-activated protein kinases (MAPKs), in particular p38 MAPK, have been implicated in autophagy signaling [27, 28]. To determine whether p38 MAPK and ERK pathways are involved in the induction of autophagy by RA + ω-3 PUFAs in breast cancer cells, we examined p38 MAPK and ERK pathways in breast cancer cells treated with RA and ω-3 PUFAs singly or in combination.

**Figure 4:**
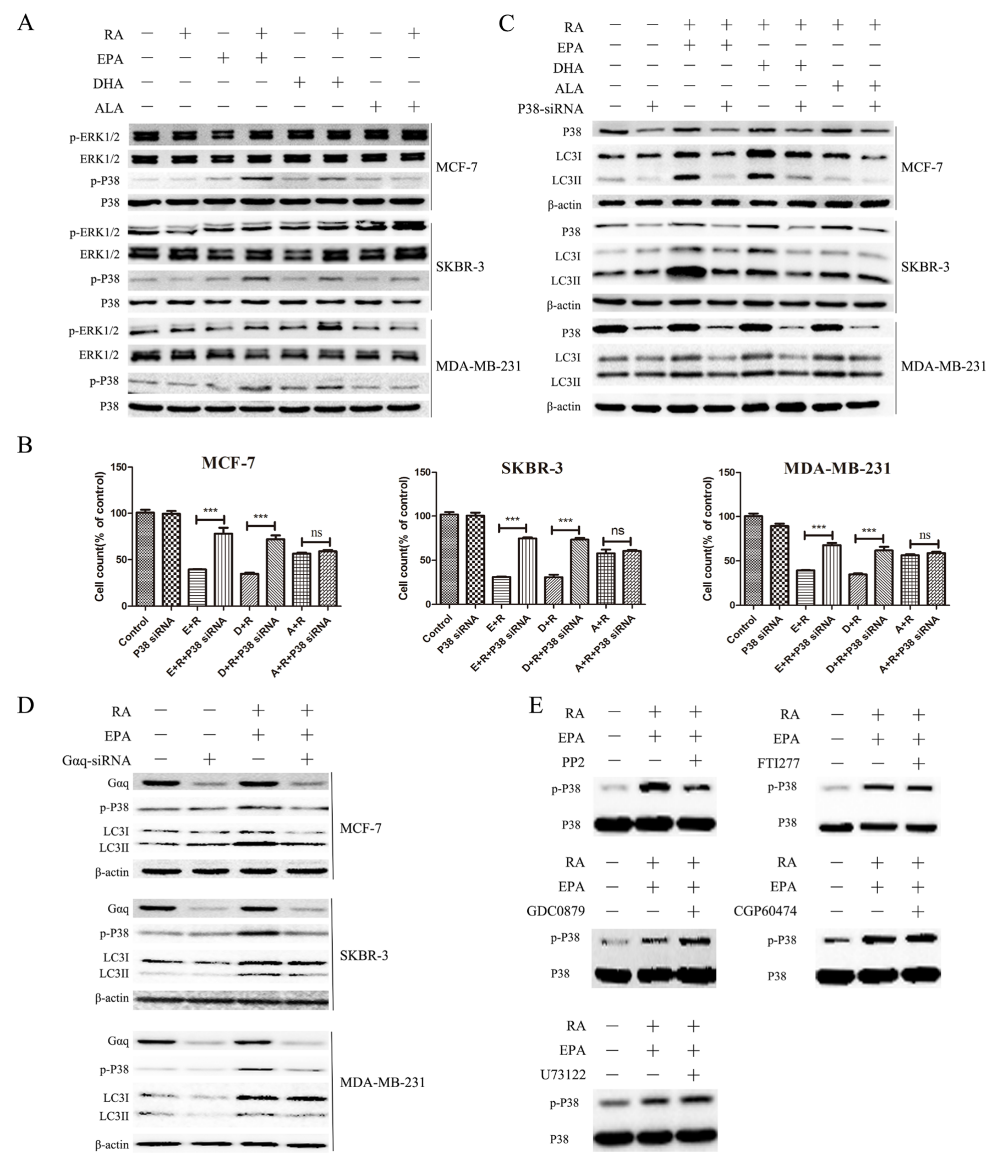
Autophagy induction by RA and ω-3 PUFA treatment is dependent on Gαq-P38 activation **(A)**: Cells were treated with RA(20μM) or ω-3 PUFAs(80μM) alone or in combination for 24h. Cell extracts were prepared and subjected to western blotting analysis. **(B)**: Cell counts of breast cancer cells treated with RA(20μM) plus ω-3 PUFAs(80μM) with or without P38-knockdown for 24h. **(C)**: Cells were treated with RA(20μM) +ω-3 PUFAs(80μM) with or withoutP38-knockdown for 24h.Cell extracts were prepared and subjected to western blotting analysis. **(D)**: Cells were treated with RA(20μM) + EPA(80μM) with or without Gαq-knockdown for 24h.Cell extracts were prepared and subjected to western blotting analysis. **(E)**: MCF-7 cells were pretreated with the indicated chemical inhibitors for 30min, followed by 15 min treatment with RA(20μM) + EPA(80μM).Cell extracts were prepared and subjected to western blotting analysis. Data are given as mean ± SEM. ^*^p < 0.05, ^**^p < 0.01, and ^***^p < 0.001.

As shown in Figure 4A, the levels of phosphorylated p38 were significantly increased in the groups treated with RA + DHA or EPA, but not in the group treated with RA + ALA, compared with the groups treated with RA or ω-3 PUFAs alone. However, No significant changes in the ERK pathway were detected in any of the groups. To further clarify whether p38 MAPK activation is involved in the induction of autophagy by RA + ω-3 PUFAs, p38 MAPK-specific siRNA was used to knock down the p38 MAPK gene. When treated with RA + ω-3 PUFAs, cells transfected with p38 MAPK siRNA showed a significant decrease in cell death compared with cells transfected with non-targeting control siRNA, except for the group treated with RA + ALA (Figure 4B). Moreover, western blot analysis indicated that P38 knockdown attenuated LC3-II accumulation induced by combination treatment (Figure 4C). These results suggest that the p38 MAPK signaling pathway was activated by combination treatment and contributed to the induction of autophagy in breast cancer cells. (Supplementary Figure 2A) However, ALA was significantly less effective than other ω-3 PUFAs in combination with RA, which may be due its low affinity for the receptor. Therefore, EPA was used in subsequent experiments.

G proteins are involved in the signal-coupling mechanisms of heptahelical cell surface receptors and play significant roles in the regulation of MAPK networks and interactions between Gαq and p38MAPK [29, 30]. Based on our results, we hypothesized that Gαq may participate in p38 activation induced by RA and ω-3 PUFAs. To confirm our hypothesis, cells were transfected with Gαq-specific siRNA. As expected, the p38 phosphorylation levels and LC3II/β-actin in cells treated with RA + EPA were significantly reduced in all of breast cancer lines tested (Figure 4D).

The PLC-DAG-PKC signaling pathway of Gαq (in addition to the activation of Src or an Src-like tyrosine kinase) has been shown to be involved in the Gαq-mediated activation of p38MAPK [31]. Thus, we used a variety of inhibitors commonly used for signal transduction studies to determine which kinase participated in the Gαq-mediated activation of p38. Pretreatment with PP2 (a Src family kinase inhibitor) prevented p38 MAPK phosphorylation (Figure 4E). This indicated that Gαq-induced p38MAPK activation was dependent on Src family kinases.

### RARα and GPR40 activation contributes to Gαq-mediated p38 phosphorylation

**Figure 5:**
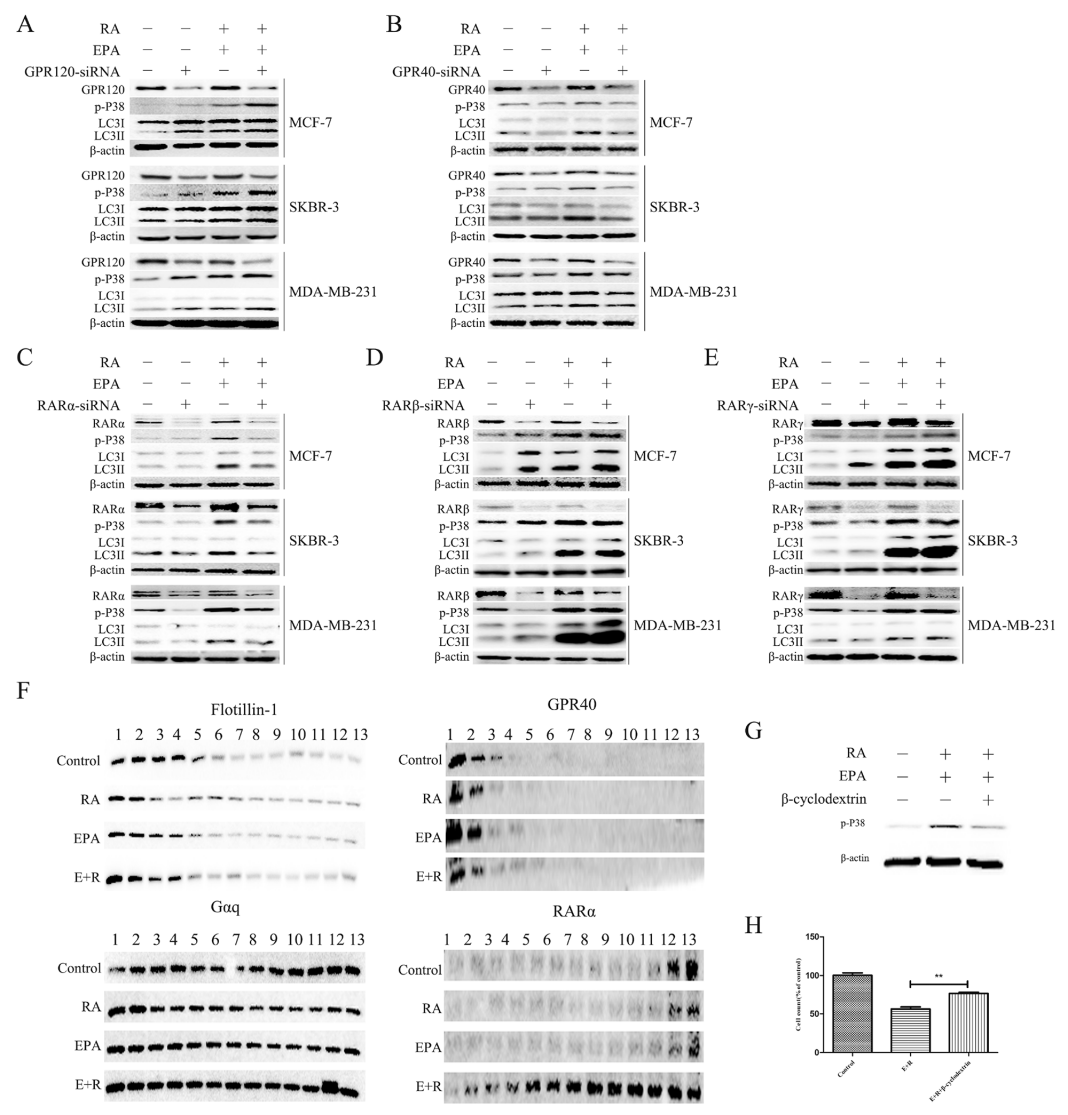
The combination of RA and ω-3 PUFAs induces Gαq-P38 activation through RARα and GPR40 **(A)**: Cells were treated with RA(20μM) + EPA(80μM) with or without GPR120-knockdown for 15 min. Cell extracts were prepared and subjected to western blotting analysis. **(B)**: Cells were treated with RA(20μM) + EPA(80μM) with or without GPR40-knockdown for 15 min. Cell extracts were prepared and subjected to western blotting analysis. **(C)**: Cells were treated with RA(20μM) + EPA(80μM) with or without RARα-knockdown for 15 min. Cell extracts were prepared and subjected to western blotting analysis. **(D)**: Cells were treated with RA(20μM) + EPA(80μM) with or without RARβ-knockdown for 15 min. Cell extracts were prepared and subjected to western blotting analysis. **(E)**: Cells were treated with RA(20μM) + EPA(80μM) with or without RARγ-knockdown for 15 min. Cell extracts were prepared and subjected to western blotting analysis. **(F)**: MCF-7 cells were treated with RA(20μM) + EPA(80μM) and their extracts fractionated using an iodixanol density gradient, as described in the Materials and Methods section. Each fraction was subjected to SDS-PAGE and immunoblot analysis using antibodies against the indicated proteins. **(G)**: MCF-7 cells were pretreated with the indicated concentrations of methyl-β-cyclodextrin (MβCD) for 1 h, followed by 15 min treatment with RA(20μM) + EPA(80μM). Cell lysates were prepared and subjected to SDS-PAGE and immunoblot analysis. **(H)**: MCF-7 cells were pretreated with the indicated concentrations of methyl-β-cyclodextrin (MβCD) for 1 h followed by 24h treatment with RA(20μM) + EPA(80μM), and then subjected to cell counts.

Previous studies have shown that GPR120 and GPR40 are cell membrane receptors for ω-3 PUFAs. To determine whether GPR40 and GPR120 transduce ω-3 PUFAs signals and activate p38, we performed knockdown experiments in breast cancer cells. The results are shown in Figure 5A and 5B. Downregulation of GPR40 gene expression significantly decreased combination-treatment-induced p38 phosphorylation and LC3II/β-actin levels in all three of the breast cancer cell lines. Suggesting that a positively regulatory effect of GPR40 on p38 activation. Conversely, p38 phosphorylation was significantly elevated by knockdown of GPR120, indicating that GPR120 downregulates p38 activity.

The bio-activity of RA is primarily mediated by members of the retinoic acid receptor (RAR) subfamily, namely RARα, RARβ, and RARγ. These belong to the nuclear receptor (NR) superfamily of transcription factors [1]. Previous studies have shown that RA mainly exhibits antitumor activity via binding to RARα [32, 33]. Moreover, it was recently reported that RA-induced autophagy in breast cancer occurred through the activation of RARα [17]; although we did not detect any significant changes in autophagy-related proteins in cells treated with RA alone. To determine which RA receptor participated in the combination treatment, specific siRNAs were used to knock down the RARα, RARβ and RARγ genes. Downregulation of RARα expression reduced the levels of combination-induced p38 phosphorylation and LC3II/β-actin levels compared with those of the RA-resistant and RA-sensitive control groups. However, this was not the case when RARβ and RARγ expression were downregulated. These results suggested that RA binds to the RARα receptor, thereby promoting p38 activation and autophagy induction. (Supplementary Figure 2B-2F) Collectively, these data highlight the importance of the co-activation of RARα and GPR40 for p38 activation in response to RA and ω-3 PUFAs treatment.

Evidence suggests that GPCRs and other relevant signaling molecules preferentially partition to highly-organized cell membrane micro-domains that are enriched with cholesterol, sphingolipids, and saturated acyl chains (lipid rafts). Hence, lipid rafts are crucial for the trafficking and signaling of GPCRs [34]. Furthermore, recent reports have indicated that RARα can present in membrane lipid rafts and form complexes with Gαq after RA stimulation, and that the formation of RARα/Gαq complexes is suppressed in RA-resistant breast cancer cells [11]. To confirm whether a contact or structural change occurs between GPR40 and RARα in lipid rafts after treatment with RA + EPA, we isolated these membrane sub-fractions and determined the locations of GPR40 and RARα. MCF-7 cells were disrupted and their lipid rafts isolated by exploiting their high buoyancy when centrifuged on a discontinuous iodixanol density gradient. After treatment with RA + EPA, RARα expression in MCF-7 cells increased significantly in the whole fractions, and the majority of RARα transferred from cytoplasm to lipid rafts (Figure 5F). Moreover, RARα was detected in the same fractions as GPR40. To confirm whether p38 phosphorylation and cell death induced by RA + ω-3 PUFAs were dependent on the changes in the lipid rafts, we used β-cyclodextrin to disrupt the structure of lipid rafts. The results showed that β-cyclodextrin significantly inhibited p38 activation and cell death in cells treated with RA + EPA. In summary, the localization of RARα in lipid rafts was crucial for the efficacy of the combination treatment.

### Combination treatment with RA and ω-3 PUFAs suppresses tumor growth *in vivo*

**Figure 6:**
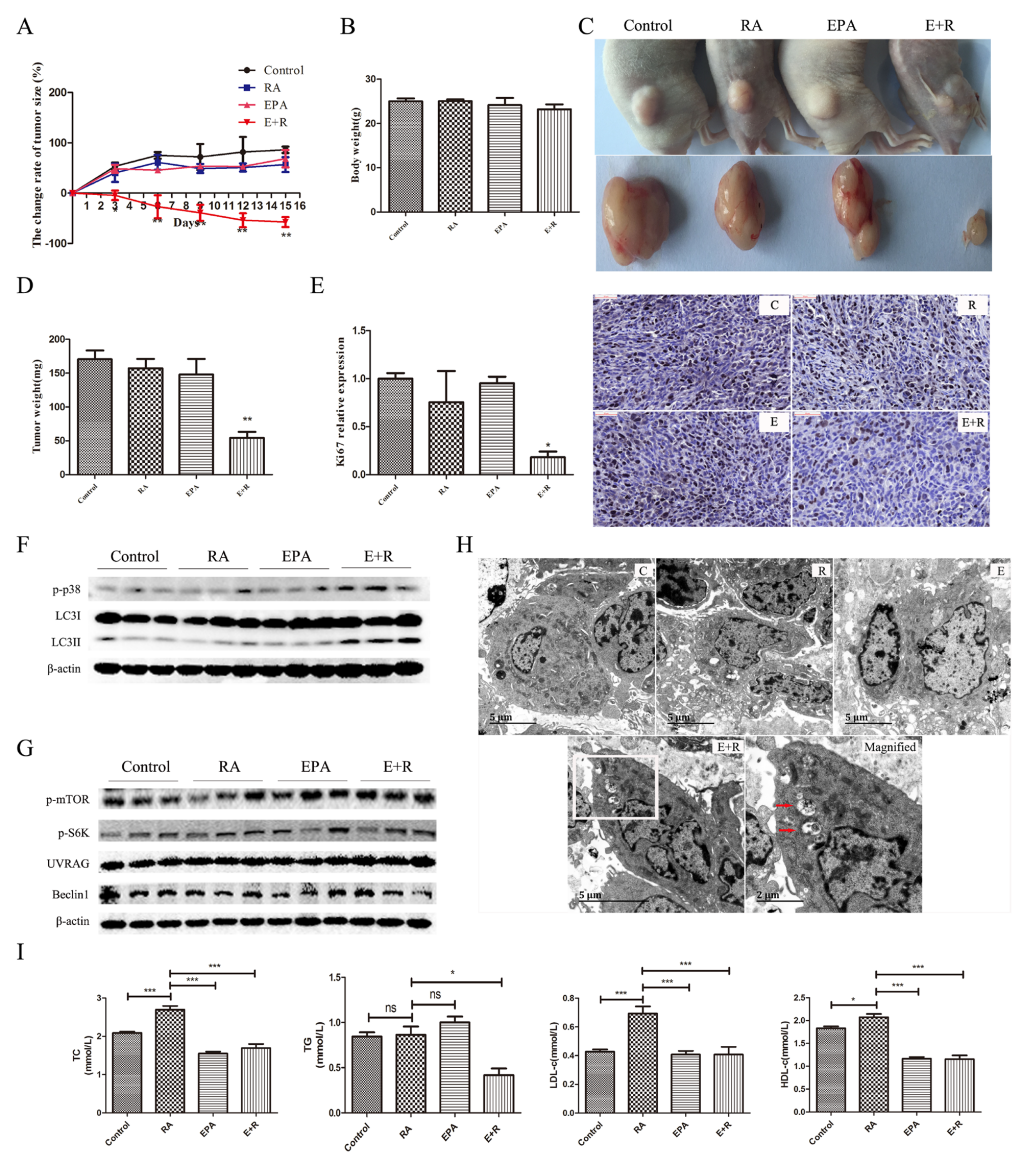
The effects of RA and EPA alone or in combination on tumor growth *in vivo* **(A)**: Effects of ω-3 PUFA and RA treatments on tumor growth in a xenograft model. Tumors were measured at the indicated time points. N=10 per group. **(B)**: Effects of ω-3 PUFA and RA treatments on body weight. **(C)**: Typical size and macroscopic appearance of tumors. **(D)**: Tumor weight in each group. **(E)**: Representative images of IHC staining of Ki67 and Ki67-positivecell numbers. The results represent the mean percentage of Ki67-positive cells relative to that of the control. **(F-G)**: Western blot analysis of xenograft models treated with RA and EPA alone or in combination. **(H)**: Tumor tissues were fixed in 2% glutaraldehyde solution and were then examined by TEM. Electron micrographs of control (C), RA treated (R), EPA treated (E), and RA + EPA treated (E+R) tissues. Magnified images of the boxed regions of d showing autolysosomes or autophagosomes (magnified) (red arrows). **(I)**: TG, TC, HDL-c, and LDL-c levels were determined by a programmable automatic biochemical analyzer (Mindray, BS480, China) according to the manufacturer’s instructions. Data are given as mean ± SEM. ^*^p < 0.05, ^**^p < 0.01, and ^***^p < 0.001.

To evaluate the anticancer efficacy of the combination treatment on breast cancer *in vivo*, we used MDA-MB-231 cancer cell xenografts in athymic nu/nu mice as a representative *in vivo* model. Neither RA nor EPA significantly inhibited tumor growth when administered singly, compared with the control group (Figure 6A). These results were in agreement with previous reports [35, 36]. However, the combination treatment caused significant reductions in tumor growth in a time-dependent manner. At the end of the treatment period, the average tumor volume in the combination treatment group was significant smaller than that of the other groups (Figure 6C). Moreover, the tumor mass showed the same trend (Figure 6D). As retinoic acid and ω-3 fatty acids are safety in the course of clinic application, we did not investigate the toxicity of combinational treatment in our experiment strictly, but measured the change of body weight in each group and we did not find significant change of body weight (Figure 6B).

A number of studies have confirmed that Ki67 protein expression is a strong indicator of patient outcome [37]. Therefore, it was necessary to determine Ki67 expression in each group. Tumor tissues were collected and Ki67 expressions were assessed using immunohistochemistry (IHC). A significant decrease in Ki67 expression was observed following treatment with RA + EPA. The RA-treated group exhibited anon-significant decrease in Ki67 expression, and EPA alone had no effect on Ki67 expression (Figure 6E). To determine whether the cell death mechanism observed *in vitro* could also be detected *in vivo*, key proteins in tumor tissues were analyzed. The *in vivo* data strongly supported *in vitro* data; treatment with RA + EPA significantly increased LC3II/β-actin expression associated with p38 activation (Figure 6F). Protein expression related to mTOR and Beclin-1 complexes did not change significantly in mice that received this combination treatment (Figure 6G). This was also in agreement with our *in vitro* results. Furthermore, TEM showed that the quantity of autophagosomes was significantly greater in the combination treatment group (Figure 6H) than in the singly-treated and control groups. These data further support the proposed therapeutic mechanism of ω-3 PUFA-supplemented RA treatment.

Clinical investigations have shown that metabolic abnormalities consistently occurred in patients during treatment with RA. The incidence of hypertriglyceridemia and hypercholesterolemia were about 44 and 31%, respectively [38]. To explore whether EPA supplementation could improve RA-induced metabolic disorders, serum biochemical indexes were measured, including TG, TC, HDL-c, and LDL-c. EPA was found to completely correct RA-induced hypercholesterolemia. However, in our xenograft model, no significant changes were observed in serum TG between RA-treated mice and other groups.

## DISCUSSION

RA is the first clinically useful cyto-differentiating agent, and is used in the treatment of acute promyelocytic leukemia (APL)[39]. Presently, there is interest in extending the therapeutic uses of RA and its derivatives to breast cancer. The large number of pre-clinical studies of RA translated into a small number of clinical trials. The only RA-based trial was a phase-II study in pre-treated patients which failed to achieve the primary end-point. The low effectiveness of RA in breast cancer therapy may be attributed to RA-induced metabolic dysfunction and drug resistance, especially in TNBC. Therefore, it is unlikely that RA will ever be an effective breast cancer therapy when used as a single agent, and that the development of RA-based combination treatments is important.

**Figure 7:**
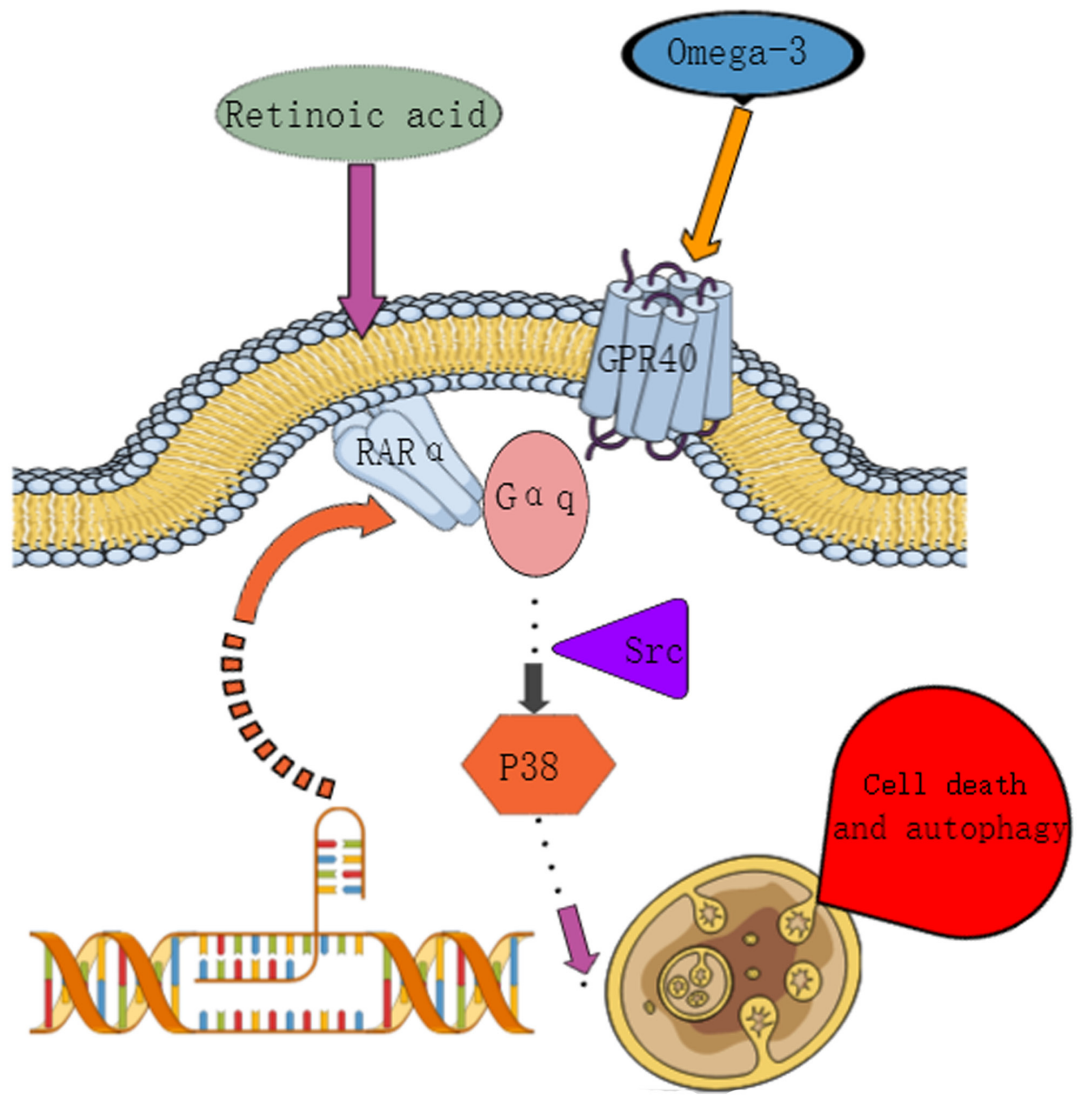
Signal transduction pathway

In this study, we found that the combination of low-doses of RA and ω-3 PUFAs selectively induced non-canonical autophagy *in vitro* and *in vivo*through a GPR40 and RARα co-activation mediated Gαq-p38MAPK pathway. ω-3 PUFAs potentiated the efficacy of RA and restored RA sensitivity in three different breast cancer subtypes. Furthermore, supplementation with ω-3 PUFAs significantly improved RA-induced hypercholesterolemia (Figure 7).

Although apoptotic cells were observed in 72h after RA and ω-3 PUFAs treatment (data not shown), cell apoptosis did not illustrate the synergistic effect of cell growth inhibition by combined treatment because of the rapid cell death and morphological change. The most possible cause is autophagy induction. Autophagy is characterized by massive degradation of cellular contents, intracellular membrane/vesicle reorganization, and lysosomal activity. Moreover, studies have demonstrated that autophagy is often initiated significantly earlier than apoptosis [40, 41]. Thus, our experiments focused mainly on autophagy. Multiple classical methods (western blot, TEM, and autophagic flux) confirmed that this combination treatment induced autophagy. Moreover, the xenograft model study demonstrated the therapeutic effect of RA + EPA treatment. Additionally, autophagy was induced by RA +ω-3 PUFAs treatment independent of the two major protein complexes (mTOR1and Beclin-1) responsible for the initiation and nucleation of autophagosome formation.

Subsequently, we found that p38MAPK participated in autophagy activation induced by RA +ω-3 PUFAs combination treatment. Although previous studies [11, 42] have shown that RA-induced apoptosis or differentiation were dependent on p38 activation, we did not detect any significant change in p38 phosphorylation when RA was used as a single treatment agent. This may be due to the use of different check points. In some studies, p38 phosphorylation was examined at 24h and 48h after stimulation, while in other studies it was examined after 10–30min. Our results suggested that ω-3 PUFAs increased the duration of RA-induced p38 activation and that it was sustained p38 activation that led to cell death.

Recent research has demonstrated that stimulation by both ω-3 PUFAs and RA resulted in Gαq activation [11, 43]. Thus, Gαq was a common target of ω-3 PUFAs and RA. Hence, we performed knockdown studies on Gαq to confirm this hypothesis. We found that p38 phosphorylation and LC3II/β-actin levels induced by combination treatment was impaired after Gαq knockdown. In addition, we used several kinase inhibitors related to Gαq-mediated p38 phosphorylation to determine which kinase was involved in Gαq-mediated MAPK activation. Our results demonstrated that Src family kinases were involved in p38 MAPK phosphorylation [29].

The biological activity of classic retinoids is primarily mediated by nuclear retinoid receptors, which are divided into RARs (RARα, RARβ and RARγ) and RXRs (RXRα, RXRβ and RXRγ). RA is a pan-RAR agonist capable of activating all RAR-isoforms with similar efficiencies [44]. Prior reports have indicated that GPR120 and GPR40 participate in ω-3PUFA-mediated signal transduction [9, 10]. However, there are disagreements between these studies regarding which specific receptor was involved in such regulation. Therefore, it is necessary to identify the specific receptors of RA and ω-3 PUFAs involved in Gαq-p38 activation induced by the combination treatment. Our results indicated that RARα and GPR40 were the key receptors mediating Gαq-p38 activation. Most previous studies determined that the beneficial effects of ω-3 PUFAs were dependent on GPR120at early time points [45, 46]. However, a recent study revealed that the reduction of tumor growth by ω-3 PUFAs was not dependent on GPR120 [36]. This agrees with the results of the present study(in which GPR40 was the receptor responsible for transferring ω-3 PUFA signals to cytoplasm).

We hypothesized that the significant changes induced by RA + ω-3 PUFA treatments were the result of a more complex process than simple signal potentiation. Signaling pathways are initiated at the plasma membrane in lipid rafts and amplified by activation cascades of downstream effectors, including G proteins. Moreover, a study found that RA can induce the formation of RARα-Gαq complexes in lipid rafts [11]. This prompted us to investigate whether the combination treatment caused changes in lipid rafts. We determined that RA + EPA treatment significantly increased the expression of RARα in whole fractions, and that a significant proportion of the RARα transferred from cytoplasm to lipid rafts (Figure 5F). Moreover, RARα was detected in the same fractions as GPR40, suggesting that RARα and GPR40 may form a complex in lipid rafts, but this conclusion needs more experiments to demonstrate. A considerable amount of literature has been published concerning the role of RARα downregulation in drug resistance [47, 48]. Therefore, increased RARα expression may be the key determinant in restoring RA sensitivity in MDA-MB-231.

Numerous studies have demonstrated that ω-3 fatty acids play important roles in a variety of cellular processes and have been shown to reduce circulating triglycerides, cholesterol-containing remnant lipoproteins, oxidized LDL-C (ox-LDL-C) [49]. EPA as a representative of ω-3 fatty acids has more powerful function in improving lipid and cholesterol metabolism. Studies have found EPA can significantly inhibit the oxidation of apolipoprotein B (ApoB)-containing lipid particles, including LDL, small dense low-density lipoprotein (sdLDL), and very low-density lipoprotein (VLDL) [50]. In addition, treatment with EPA resulted in a dose-dependent reduction in cholesterol domain formation. EPA corrected RA-induced hypercholesterolemia may through inhibit the oxidation of apolipoprotein B (ApoB)-containing lipid particles and suppress cholesterol domain formation.

In conclusion, this study demonstrated a novel combination of RA and ω-3 PUFAs for breast cancer treatment. This combination is characterized by its low toxicity and strong therapeutic effect. However, further experiments are necessary to determine the dispersion of RARα on sub-membrane lipid rafts and for elucidating the molecular mechanism of the synergistic effect of RARα and GPR40 in the induction of autophagy. Further characterization of this synergistic effect and that of RA andω-3 PUFAs will provide unique insights into the molecular mechanisms of cell autophagy flux and provide better therapeutic targets for breast cancers, particularly in patients who develop resistance to RA therapy.

## MATERIALS AND METHODS

### Drugs and reagents

RA, 3-MA, Rapamycin, Chloroquine (CQ), PP2, FTI277, U73122, GDC0879, and CGP60474 were purchased from MedChemExpress(China). DHA, EPA, and ALA were purchased from Nuchek (USA). Insulin was purchased from Sigma Aldrich (USA).

### Cell culture

MCF-7, SK-BR-3, and MDA-MB-231 (Institute of Cell Biology, Shanghai, China) were maintained in DMEM media (Gibco, USA), and supplemented with 10% FBS (Gibco, USA), 100μg/ml penicillin, and 100 μg/ml streptomycin, respectively, in a humidified atmosphere containing 5% CO_2_ at 37°C. Prior to treatment, cells were grown to 60–70% confluence and exposed to serum-free medium for 24 h.

### Cell viability assay

CCK8 assay and cell counting method were performed to evaluate cell viability. Cell Counting Kit 8 (CCK8) was purchased from Dojindo Molecular Technology (Tokyo, Japan). For CCK8 assay, cells were cultured in 96-well plates at a density of 5000 cells per well in 100μl medium. ω-3 FFAs, RA and the combination were added into the wells and incubated for 72h. Then, cells were added 10μl CCK8 substrate and incubated for another 3 h at 37°C. The optical density was measured at 450 nm on a microplate reader Multi-skan GO (Thermo Scientific, USA). For cell counting method, cells were cultured in 6-well plates and treated in the same way. Then, cells were digested by trypsin and then counted by blood platelet count.

### siRNA transfection and western blot analysis

P38, Gαq, GPR120, GPR40, RARα, RARβ, and RARγ siRNA oligos were obtained from Gene Pharma (Suzhou, China) and used as non-targeting controls. Cells were transfected with appropriate siRNAs using jetPRIME reagent (Polyplus) according to the manufacturer’s protocol. Cells were harvested, washed twice with ice-cold phosphate-buffered saline (PBS), and subjected to western blot analysis, each western blot was measured two times as described previously [51]. The antibodies used were as follows. LC3B(#L7543) was purchased from Sigma-Aldrich. mTOR(#A2445), phospho-mTOR(S2448,#AP0094), Beclin-1(#A7353), and UVRAG(#A8462) were purchased fromAbclonal. phospho-p70S6-kinase(T389,#9234), phospho-p38(Thr180/Tyr182,#4511), phospho-ERK1/2(Thr202/Tyr204,#4370), p38(#8690), ERK1/2(#4695), and Gαq(#14373) were purchased from Cell Signaling Technology. RARα(C20,#sc-551), RARβ(C19,#sc-552), RARγ(C19,#sc-550), GPR40(FL300, sc-32905) and β-actin (N21,#sc-130656)were purchased from Santa Cruz Biotechnology. GPR120(#NBP1-00858) was purchased from NovusBiologicals. Ki67(#ab15580) and Flotilin-1 (ab133497) were purchased from Abcam. Goat anti-rabbit (A00098) secondary antibody was purchased from Genescript.

### Isolation of lipid rafts

Membrane lipid rafts were isolated using the procedure described by Ostrom and Insel [52]. Briefly, 6 × 10^7^ cells were grown in petri dishes, washed twice with PBS and lysed for 1 min on ice in 500μl of 10mMTris-HCl (pH 7.4) containing 1% (v/v) Triton X-100, 1 mM EDTA, and protease/phosphatase inhibitors. The cell lysate was transferred to a 2-ml Dounce homogenizer and homogenized (10 strokes) on ice. The lysate was adjusted to2ml. An equal volume of 50%iodixanolwas added to the lysate and mixed thoroughly by pipetting up and down several times to give a final concentration of 25%iodixanolin 4ml of lysate. Then, 400μl of 15% iodixanol, 400μlof 5% iodixanol, and 400μl of 0% iodixanol were carefully layered on the lysate. After centrifugation at160,000g for 4h at 4°C, fractions (13 in total) were collected from the top of the tube. Fractions were analyzed by SDS–PAGE and immunoblotting assays.

### Transmission electron microscopy

Transmission electron microscopy (TEM) was used for ultrastructural analysis. After treatment, cells were fixed and embedded. Sections (90 nm) were cut and examined by TEM at 80 kV (Hitachi HT7700).

### Xenograft breast cancer model and treatment

The animal protocol was approved by the Jiangnan University Animal Care and Use Committee. Female nude mice (purchased from Slaccas, Shanghai, China)aged 4 to 5 weeks received injections of 4 × 10^5^ breast cancer cells(MDA-MB-231). Two weeks later, mice with similar tumor volumes (200mm^3^) were randomized into 4treatment groups: control (vehicle), EPA(3g in 100g diets), RA(5mg in 100g diets), and EPA and RA in combination. The mice were treated for 2 weeks. Tumor sizes were measured twice a week. The tumor volumes were calculated using the following formula: volume =½ × length × width^2^. The mice were sacrificed after the treatment period. Blood was collected from the retroorbital sinus of each non-anesthetized mouse for measuring biochemical indexes, including serum triglyceride (TG), cholesterol (TC), HDL-c, and LDL-c. Analyses were conducted using a programmable automated biochemical analyzer (Mindray, BS480, China) according to the manufacturer’s instructions. Paraformaldehyde-fixed tumor tissues were embedded in paraffin and sectioned (4 mm). The sections were treated with 0.3% hydrogen peroxide/methanol and incubated with monoclonal antibodies, followed by incubation with Histostain-Plus IHC Kit reagents according to the manufacturer’s instructions.

### Statistical analysis

All experiments were performed in triplicate. Data were reported as mean ± SEM. Statistical significance was determined by one-way analysis of variance (ANOVA) followed by Dunnett’s test for multiple comparisons. Statistical analyses were performed using SPSS software (version 20.0; SPSS Inc., USA). ^*^p <0.05, ^**^p < 0.01, and ^***^p < 0.001.

## SUPPLEMENTARY MATERIALS FIGURES


