# Supplementary material for: RA and ω-3 PUFA co-treatment activates autophagy in cancer cells
Source: Oncotarget. 2017 Nov 22;8(65):109135–50. doi: 10.18632/oncotarget.22629 (PMC5752509; doi:10.18632/oncotarget.22629)
Supplement: Supplementary file 1 [file oncotarget-08-109135-s001.pdf]

RA and ω-3 PUFA co-treatment activates autophagy in cancer cells

SUPPLEMENTARY MATERIALS

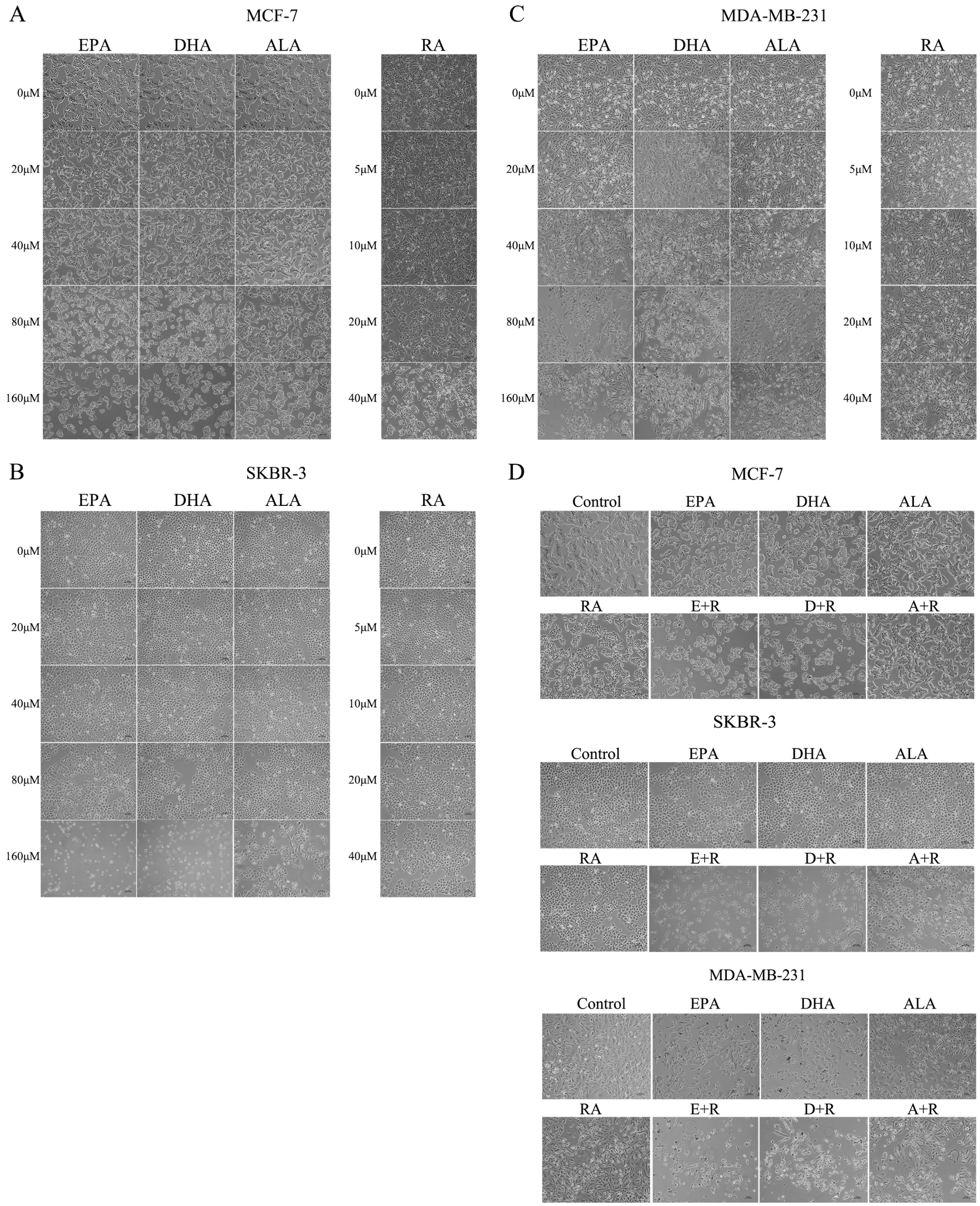

Supplementary Figure 1: Cell morphology of three breast cancer cell lines treated with RA and ω-3 PUFAs. (A) Cell morphology of MCF-7. **(B)** Cell morphology of SKBR-3. **(C)** Cell morphology of MDA-MB-231. **(D)** Cell morphology after combination treatment.

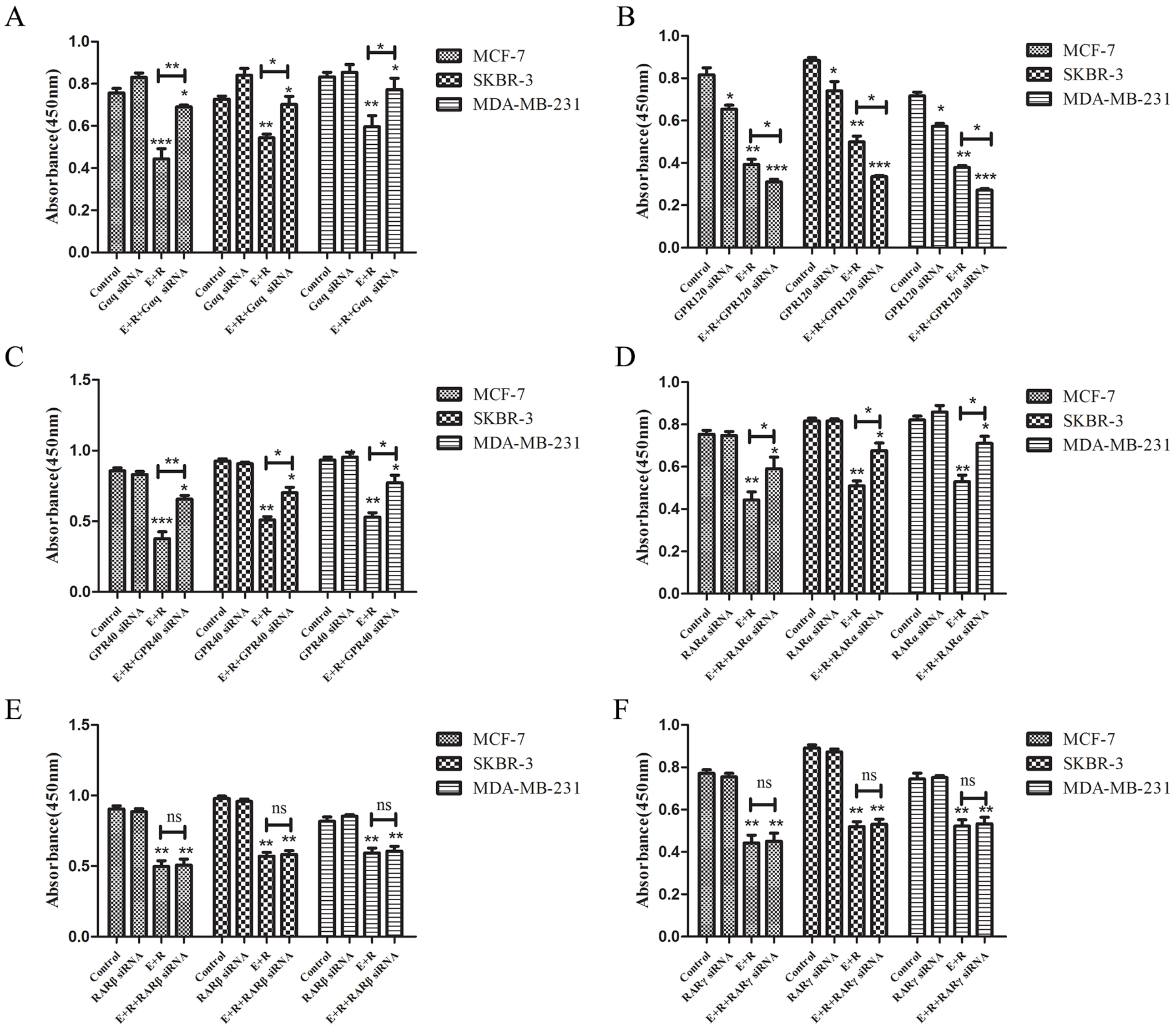

Supplementary Figure 2: Cell viability of breast cancer cells treated with RA plus EPA with or without genes knockdown. (A): Cell viability of breast cancer cells treated with RA plus EPA with or without Gαq knockdown for 24h. **(B)**: Cell viability of breast cancer cells treated with RA plus EPA with or without GPR120 knockdown for 24h. **(C)**: Cell viability of breast cancer cells treated with RA plus EPA with or without GPR40 knockdown for 24h. **(D)**: Cell viability of breast cancer cells treated with RA plus EPA with or without RARα knockdown for 24h. **(E)**: Cell viability of breast cancer cells treated with RA plus EPA with or without RARβ knockdown for 24h. **(F)**: Cell viability of breast cancer cells treated with RA plus EPA with or without RARγ knockdown for 24h.
